# Vitamin e-loaded membrane dialyzers reduce hemodialysis inflammaging

**DOI:** 10.1186/s12882-019-1585-6

**Published:** 2019-11-15

**Authors:** Vincenzo Sepe, Marilena Gregorini, Teresa Rampino, Pasquale Esposito, Rosanna Coppo, Francesco Galli, Carmelo Libetta

**Affiliations:** 1 Unit of Nephrology and Dialysis, Transplantation; Fondazione IRCCS Policlinico «San Matteo», Viale Camillo Golgi 19, 27100 Pavia, Italy; 2 UOC di Nefrologia e Dialisi, Trapianto, Fondazione IRCCS Policlinico «San Matteo», Viale Camillo Golgi, 19, 27100 Pavia, Italy; 3 Chair of Nephrology, University of Pavia, Corso Strada Nuova 65, 27100 Pavia, Italy; 4 Fondazione Ricerca Molinette, Regina Margherita Hospital 94, Piazza Polonia, 10126 Torino, Italy; 5 Università degli Studi di Perugia, Pharmaceutical Sciences, Branch of Via del Giochetto, building B, 2nd floor, 06123 Perugia, Italy

**Keywords:** Biocompatibility, Indoleamine 2,3−dioxygenase, Inflammaging, Nitric oxide, Vitamin E−loaded multi−layer hemodialysis filter

## Abstract

**Background:**

Inflammaging is a persistent, low−grade, sterile, nonresolving inflammatory state, associated with the senescence of the immune system. Such condition downregulates both innate and adaptive immune responses during chronic disorders as type II diabetes, cancer and hemodialysis, accounting for their susceptibility to infections, malignancy and resistance to vaccination.

Aim of this study was to investigate hemodialysis inflammaging, by evaluating changes of several hemodialysis treatments on indoleamine 2,3-dioxygenase-1 activity and nitric oxide formation.

**Methods:**

We conducted a randomized controlled observational crossover trial. Eighteen hemodialysis patients were treated with 3 different hemodialysis procedures respectively: 1) Low−flux bicarbonate hemodialysis, 2) Low−flux bicarbonate hemodialysis with vitamin E − loaded dialyzers, and 3) Hemodialfitration. The control group consisted of 14 hospital staff healthy volunteers. Blood samples were collected from all 18 hemodialysis patients just after the long interdialytic interval, at the end of each hemodialysis treatment period.

**Results:**

Hemodialysis kynurenine and kynurenine/L − tryptophan blood ratio levels were significantly higher, when compared to the control group, indicating an increased indoleamine 2,3-dioxygenase-1 activity in hemodialysis patients. At the end of the low−flux bicarbonate hemodialysis with vitamin E − loaded dialyzers period, L − tryptophan serum levels remained unchanged vs both low−flux bicarbonate hemodialysis and hemodialfitration. Kynurenine levels instead decreased, resulting in a significant reduction of kynurenine/L − tryptophan blood ratio and indoleamine 2,3-dioxygenase-1 activity, when matched to both low−flux bicarbonate hemodialysis and HDF respectively. Serum nitric oxide control group levels, were significantly lower when compared to all hemodialysis patient groups. Interestingly, low−flux bicarbonate hemodialysis with vitamin E − loaded dialyzers nitric oxide serum levels from venous line blood samples taken 60 min after starting the hemodialysis session were significantly lower vs serum taken simultaneously from the arterial blood line.

**Conclusions:**

The treatment with more biocompatible hemodialysis procedure as low−flux bicarbonate hemodialysis with vitamin E − loaded dialyzers, reduced indoleamine 2,3-dioxygenase-1 activity and nitric oxide formation when compared to both low−flux bicarbonate hemodialysis and hemodialfitration. These data suggest that low−flux bicarbonate hemodialysis with vitamin E − loaded dialyzers lowering hemodialysis inflammaging, could be associated to changes of proinflammatory signalling a regulated molecular level.

**Trial registration:**

NCT Number: NCT02981992; Other Study ID Numbers: 20100014090. First submitted: November 26, 2016. First posted: December 5, 2016. Last Update Posted: December 5, 2016.

## Background

Inflammaging is a new field of clinical research orientated to study age–related persistent, low−grade, sterile and non−resolving inflammatory states in chronic diseases such as type II diabetes, obesity and other metabolic disorders [1]. Such clinical states are mainly sustained by macrophage proinflammatory activation. Increased expression of IDO1 appears to modulate macrophage differentiation towards the M2 phenotype [2]. Nevertheless, recent studies suggest a deficit of macrophage polarization capacity in aged mice [3]. Although an M2–like macrophage shift is predictive of successful biomaterial implant, it was observed a local M1 skewed host response at the site of effective of polypropylene mesh implants in aged experimental animals [3]. Data on macrophage polarization during hemodialyzer blood interaction, are not currently available. The known detrimental effect of macrophage triggering during chronic hemodialysis [4] is also associated to the increase of blood NO levels, reducing both native and adaptive immune responses [5, 6].

Uremia is characterized by chronic systemic inflammation and chronic activation of the immune system, often followed by immune deficiency [7, 8]. Impaired immune response in uremic patients is mainly characterized by innate and adaptive immune regulation with a T cell number decrease, function loss [9], and complement dysregulation [10, 11], which results in a frequent susceptibility to sepsis [12], malignancy [13], resistance to vaccination [14] and cardiovascular diseases [15]. Oxidative stress and chronic inflammation are increasingly appearing as relevant atherosclerosis and cardiovascular mortality contributors in hemodialysis patients [16]. Accumulating evidence on end−stage renal disease patients show that uremia−associated immune deficiency is associated to chronic inflammation and immunological aging [17]. IDO1 is a unique dioxygenase class member, catalysing L−Trp to its product Kyn [18]. IDO1 and Kyn have a significant function on T cell immunosuppression, T_reg_ activation, and inhibition of natural killer cells [19]. Induction of IDO1 transcription and function, associated to T_reg_ suppressive activity, is increased in chronic hemodialysis inflammation [18–20].

Although NO is a small, easily diffusible molecule by dialysis, its levels significantly increase during hemodialysis [21, 22]. It might be related to the hyperactivation of macrophage activity by both uremia and the hemodialysis procedure itself [22]. There is a growing body of evidence suggesting that hemodialysis is characterized by excessive oxidative stress status, resulting from the loss of antioxidants during hemodialysis procedures or dietary restrictions, and the accumulation of oxidative products. Deficiency of vitamin C and selenium, reduced intracellular levels of vitamin E, and the increased synthesis of reactive oxygen species and nitrogen species as NO, are relevant mechanisms associated with hemodialysis excess of oxidative stress [23].

Since 2004, vitamin E has been submitted as drug therapy for hemodialysis microinflammation [24], and solid data are available on lowering oxidative stress and inflammation obtained using VIT−E dialyzers [25, 26]. We also observed a vast T_reg_ number in hemodialysis patients, suggesting an increased immune tolerance when compared to controls [27]. However, little experimental data are available on the role that VIT−E have in regulating the immune response associated to IDO1 activity and NO formation. A monocyte−derived cell experimental model (THP−1), found alpha-tocopherol (a vitamin E analogue) to attenuate L−Trp and IDO1 related, apoptosis after induction by IFN−γ [28]. Currently, hemodialysis data on the role vitamin E on NO formation have are not available. Moreover, still controversial is the role of VIT−E hemodialysis in atherosclerosis, when complement regulation [29], oxidative stress and inflammation prevention are specific endpoints [19]. Large population−based studies associated high dietary vitamin E supplements with a lower risk of coronary heart disease. Nevertheless, interventional studies on population with large and/or maintenance hemodialysis patients, failed to show any beneficial effect of vitamin E oral intake in the development of coronary artery disease [30]. More consistent data are published on oxidative stress in the course of treatment with VIT−E, showing improved biocompatibility and reduced reactive oxygen species formation [30]. At present, papers on both blood IDO1 activity and NO formation in hemodialysis patients and related dialysis procedures have not been published. Aim of this study was to investigate the mechanisms of hemodialysis inflammaging, comparing IDO1 blood activity and NO formation in patients treated by standard low−flux polysulfone BHD, low−flux VIT−E hemodialysis, and polysulfone HDF.

## Methods

### Study design and rationale

This study was a randomized controlled observational crossover trial. Eighteen patients randomly sampled from our hemodialysis unit, have been included in the study. Exclusion criteria included recent illness (within the previous 2 months), significant anemia (Hb < 10 g/dl), autoimmune disease, current/previous (6 months) immunosuppressive treatment, but also systemic diseases such as diabetes, vasculitis, amyloidosis, rheumatic disease; HBV, HCV, HIV positivity, other active viral and/or bacterial infection, active cancer or after remission and previous transplantation.

All 18 chronic hemodialysis patients have been on a thrice−weekly hemodialysis regimen for at least 6 months, treated with standard low−flux polysulfone dialysis. In random sequence hemodialysis patients underwent a 4−hour thrice−weekly, 3−month period of a) standard low–flux bicarbonate hemodialysis with polysulfone membrane dialyzers, (BHD; FX10 Low−Flux, Helixone® membrane, ^©^Fresenius Medical Care AG & Co. KGaA, Bad Hamburg, Germany), b) HDF (pre−dilution 70 ± 12 ml/min) with polysulfone membrane dialyzers (FX100 High−Flux, Helixone® membrane, ^©^Fresenius Medical Care AG & Co. KGaA, Bad Hamburg, Germany) and c) standard low–flux VIT−E BHD (VitabranE® membrane E18, Asahi Kasei Kuraray Medical Co., Japan). Blood samples were taken at the end of the long interdialytic interval, at the beginning and at the end of each 3–month period of the study respectively. In order to evaluate the interference of the vitamin E–loaded membrane dialyzers on NO serum levels, blood samples were also taken simultaneously from arterial and venous lines at the beginning and 60 minutes after starting the same hemodialysis session and at the end of the last long interdialytic interval of each 3–month period of the study. Current therapy remained unchanged for the duration of the study. European Renal Best Practice (ERBP) guidelines for anaemia in chronic kidney disease (CKD) were applied [31]. In detail, intravenous iron supplements were administrated as sodium ferric gluconate complex, or ferric caboxymaltose were administrated at the end of a hemodialysis session according to prescription, in order to maintain ferritin blood levels ≤ 800 ng/ml and transferrin saturation > 20%. Correction of hyperparathyroidism and hypertension treatment remained unchanged throughout the study. A control group of 14 hospital staff individuals, healthy for at least 6 months, were also recruited. Participant characteristics are summarized on Table 1.

**Table 1:** Demographic and clinical characteristic of the chronic hemodialyzed patients

	CON	HD patients
N	14	18
Sex, M:F	6:8	10:8
Age, years^a^	55.6 ± 5.8	59.9 ± 7.3^b^
Time on HD, months^a^	N/A	29.4 ± 17.1
ESRD cause, N:		
Nephroangiosclerosis	N/A	7
Polycystic kidney disease	N/A	2
Interstitial nephritis	N/A	1
Chronic glomerulonephritis	N/A	3
Chronic pyelonephritis	N/A	1
Unknown origin	N/A	4

*N* number of patients, *CON* hospital staff healthy volunteers, *HD* hemodialysis; ^a^, data are M ± SD; ESRD, end-stage renal disease; ^b^, P=NS vs CON; N/A not applicable

### Chromatographic determination of kynurenine and L − tryptophan

Serum (1 ml) was deproteinized by 100 μl 30% trichloroacetic acid (Sigma−Aldrich, Italy). An amount (250 μl) of supernatant was added to 50 μl of aqueous solution 49.4 μmol/l of theophilline as per Internal Standard (IS, Sigma−Aldrich, Italy). For delayed analysis, deproteinised samples were stored at -80 °C for at least one month. Standard stock aqueous solutions (2.47 mmol/l for Kyn and 4.41 mmol/l for Trp, both from Sigma−Aldrich, Italy) were prepared and kept frozen at -80 °C. Working standard solutions were made by appropriate dilution of a standard mixture.

HPLC method issued from separation conditions for Kyn and L−Trp was developed by Zhen et al [32]. In the present method, separation was achieved on HP1100 LC system (Agilent Tecnologies S.p.A., Italy) using a column Supelco C18 LC18DB (Sigma−Aldrich, Italy) (150 mm x 4.6 mm, particles size 5 μ) by isocratic elution (30 °C, 30 minutes). Mobile phase consisted of 50 mmol/l acetic-acetate buffer pH 4,6 (Sigma−Aldrich, Italy) and Methanol HPLC grade (VWR International PBI s.r.l, Italy) (95:5 v/v) at a flow rate of 0.8 ml/min. Eluates were monitored by DAD set at λ 360 nm for Kyn and λ 275 nm for L−Trp and IS. Absorbancy at λ 220 nm and λ 302 nm were also obtained, absorbance ratios were used for identification and purity assessment of each peak. Sample injection was 50 μl.

IDO1 activity was assessed in sera as change of L−Trp and its catabolic product Kyn (Kyn/Trp ratio), simultaneously measured using an isocratic RP HPLC method with UV detection.

### Total nitric oxide assay

We investigated the effects of dialysis treatment on NO serum levels in peripheral blood. Blood samples were obtained at the end of the long interdialytic interval of the last 3−month hemodialysis treatment periods. Moreover, during the last 3−month session of each hemodialysis treatment period, at the end of the long interdialytic interval, blood specimens were also taken 60 minutes after the beginning of the same hemodialysis session, simultaneously from the arterial and venous dialysis lines. We measured NO by photometric analysis with a nitrate/nitrite colorimetric assay kit (R&D Systems, Minneapolis, MN, USA). NO production was determined as NO_2_^+^ NO_3_^-^ with the Griess reagent after reduction of nitrate, to nitrite with nitrate reductase. Although several authors showed inflammation marker and oxidative stress peaks after already 15–30 minutes from the beginning of a hemodialysis session [33, 34], we designed this study consistently with a previous paper [26] where we found a significant variation of NO serum levels for 60 minutes. Readings were at 540 nm, and baseline correction was carried out at 620 nm. The sensitivity limit of the assay was 1.35 μmol/l.

### C-reactive Protein

The CRP (n.v. ≤ 6 mg/dl) was measured by the local hospital laboratory using the Latex C-Reactive Protein immunoturbidimetric assay (Alpha Laboratories Eastleigh Hampshire, UK).

### Statistical analysis

Data are presented as count or M ± SD and were analyzed by SPSS version 19.0 (Chicago, IL, USA). ANOVA with Bonferroni analysis was performed on all dependent variables. Percentage data were compared by X^2^ test to assess p-value significance. Two-tailed tests were conducted on all comparisons, and P < 0.05 was considered significant.

## Results

Table 1 shows demographic and clinical characteristics of hemodialysis patients compared to the control group. All patients were clinically stable, and hemodialysis Kt/V was always ≥ 1.2. Any vascular access, i.e. autologous arteriovenous fistula, arteriovenous graft or venous catheter, showed patency and a regular flow of > 300 ml/min during the study.

CRP was significantly higher in hemodialysis patients (8.38 ± 7.22 mg/dl) when compared to controls (3.52 ± 1.57 mg/dl, P < 0.05). Serum CRP levels were not significantly different among hemodialysis groups: BHD 9.23 ± 5.71 mg/dl, low−flux VIT−E 8.04 ± 4.02 mg/dl, HDF 7.87 ± 4.46 mg/dl.

### IDO1 activity

Table 2 summarized the data of IDO1 activity, as Kyn, Trp blood levels and Kyn/Trp ratio in controls and all hemodialysis patients at the beginning and at the end of the study respectively. Regardless to hemodialysis procedure, the Kyn/Trp ratio was significantly higher when compared to controls (P < 0.01) showing an increased IDO1 blood activity in hemodialysis patients. A significantly increased IDO1 blood activity was also observed regardless of hemodialysis procedure or hemofilter membrane utilized when compared to controls.

**Table 2:** IDO1 activity in controls and all hemodialyzed patients at the end of the study

	CON (*N* = 14)	HD patients (*N* = 18)	P
Kyn, *μmol/l*	2.86 ± 0.81	6.62 ± 1.12	< 0.05
L − Trp, *μmol/l*	24.99 ± 3.37	18.12 ± 5.09	< 0.05
Kyn/Trp	12.74 ± 2.62	39.91 ± 4.36	< 0.01

IDO1, indoleamine 2,3-dioxygenase; CON, hospital staff healthy volunteers; HD, hemodialysis; Kyn, kynurenine; L − Trp, l-tryptophan; Kyn/Trp, Kyn/ l-Trp ratio; data are M ± SD

L−Trp levels in hemodialysis patients were significantly lower when compared to controls (P < 0.05), whereas Kyn increased significantly in hemodialysis patients vs controls (P < 0.05).

However, different hemodialysis treatments influenced IDO1 blood activity (Table 3). Treatment with VIT−E reduced significantly IDO1 activity when compared to treatment without vitamin E−loaded polysulfone membranes, regardless of BHD (P < 0.05) or HDF treatments (P < 0.05). Kyn in VIT−E hemodialysis patients was significantly lower when compared to both BHD (P < 0.05) and HDF (P < 0.05).

**Table 3:** IDO1 activity in hemodialyzed patients at the end of each trial treatment period

	BHD	VIT − E	HDF
Kyn, *μmol/l*	8.23 ± 1.57*	4.60 ± 1.63	6.81 ± 1.74*
L − Trp, *μmol/l*	17.90 ± 5.62	19.50 ± 5.42	17.22 ± 4.06
Kyn/Trp	45.81 ± 8.47*	23.58 ± 4.97	39.48 ± 6.13*

IDO1, indoleamine 2,3-dioxygenase; BHD, standard low−flux bicarbonate hemodialysis with polysulfone membrane dialyzer; VIT − E, low−flux BHD with vitamin E − loaded polysulfone membrane (Excebrane®) dialyzer; HDF, hemodiafiltration with polysulfone membrane dialyzer; Kyn, kynurenine; L − Trp, l-tryptophan; Kyn/Trp, Kyn/ l-Trp ratio; data are M ± SD; *, *P* < 0.05 vs VIT − E

**Fig. 1:**
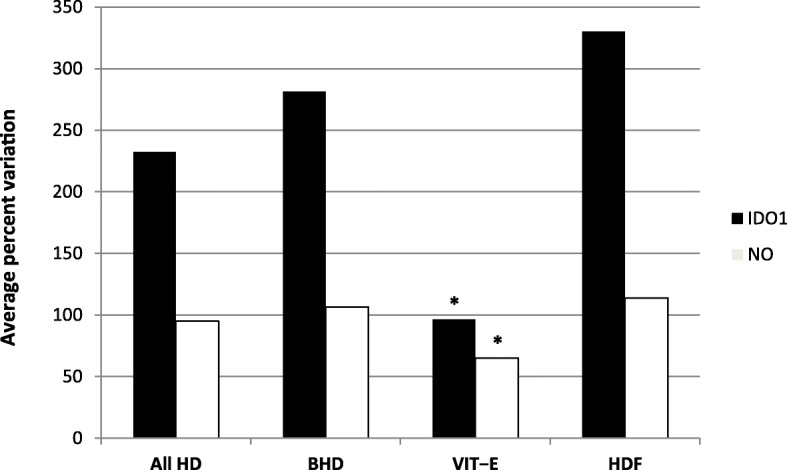
Average percent variation of blood IDO1 activity and serum NO formation in hemodialysis patients at the end of a long interdialytic interval. IDO1, indoleamine 2,3-dioxygenase serum activity (kyn/L − trp); NO, nitric oxide (μmol/l); CON, hospital staff healthy volunteers; HD, hemodialysis; BHD, standard low−flux bicarbonate hemodialysis by polysulfone membrane dialyzer; VIT − E, BHD with vitamin E − loaded polysulfone membrane (Excebrane®) dialyzer; HDF, hemodiafiltration with polysulfone membrane dialyzer; *, P < 0.05 vs All HD, BHD, HDF

Figure 1 shows percent variation of IDO1 blood activity in different hemodialysis treatments compared to controls.

### Plasma nitric oxide

**Fig. 2:**
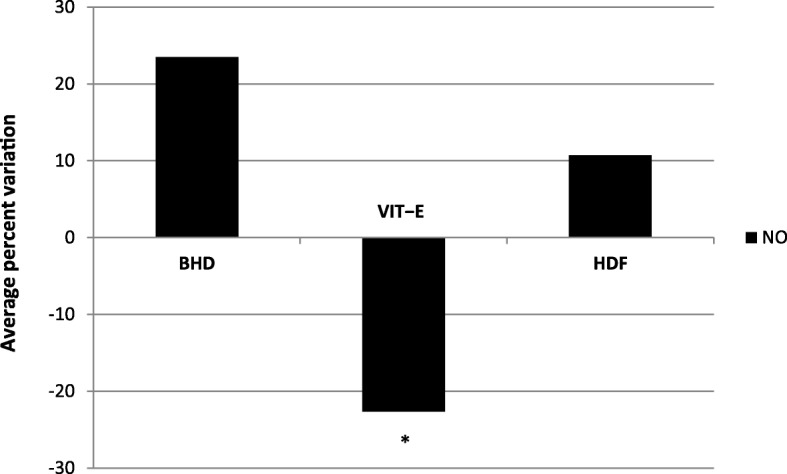
Average serum NO percent variations during the same treatment, 60 − min after the beginning of the hemodialysis session at the end of the last 3 − month period long interdialytic interval. Blood contemporarily sampled from arterial and venous blood lines. NO, nitric oxide (μmol/l); HD, hemodialysis; BHD, standard low−flux bicarbonate HD by polysulfone membrane dialyzer; VIT − E, BHD with vitamin E − loaded polysulfone membrane (Excebrane®) dialyzer; HDF, hemodiafiltration with polysulfone membrane dialyzer; *, P < 0.05 vs BHD and HDF

Data have been shown in Table 4. NO blood levels were significantly lower in controls when compared to patients under any hemodialysis procedures. NO levels were significantly higher in blood samples taken before hemodialysis treatment, in BHD and HDF when compared to VIT−E hemodialysis patients (P < 0.05). Surprisingly, NO serum levels of blood sampled from the venous line of VIT−E hemodialysis patients were significantly lower when compared to the arterial blood sampled from the same hemodialysis line. They were measured simultaneously, throughout the same treatment, at the end of the long interdialytic interval during the last VIT−E period hemodialysis session, after 1−hour VIT−E hemodialysis (Fig. 2). Percent reduction of venous vs arterial line was -22.6%. On the other hand, both BHD and HDF NO concentrations in venous hemodialysis line increased compared to arterial hemodialysis line.

**Table 4:** NO serum levels (μmol/l) in controls and hemodialyzed patients at the end of a 3 − month period long interdialytic interval HD session. During the same hemodialysis treatment blood was also drawn simultaneously, 60 min after the beginning, from both arterial and venous blood lines

	End long interdialytic interval^#^	60 − min Arterial HD line	60 − min Venous HD line
CON	40.3 ± 15.5^	N/A	N/A
BHD	88.4 ± 24.7*	85.2 ± 26.9*	105.2 ± 34.3
VIT-E	70.6 ± 16.9	65.4 ± 21.6**	50.6 ± 32.4
HDF	91.5 ± 38.0*	87.7 ± 31.2*	97.1 ± 42.8

NO, nitric oxide; HD, hemodialysis; ^#^, values at the beginning of the last HD session after a 3 − month trial period; 60 − min arterial HD line, NO serum levels from blood sampled from the arterial HD blood line 60 min after the beginning of a long interdialytic interval HD session at the end of the 3 − month trial period; 60 − min venous HD blood line, NO serum levels from blood taken from the venous HD blood line contemporarily drawn 60 min after the beginning of the last 3 − month period long interdialytic interval HD session; CON, hospital staff healthy volunteers; N/A, not applicable; BHD, standard low−flux bicarbonate HD with polysulfone membrane dialyzer; VIT − E, BHD with vitamin E − loaded polysulfone membrane (Excebrane®) dialyzer; HDF, hemodiafiltration with polysulfone membrane dialyzer; ^, *P* < 0.01 vs all HD treatments; *, *P* < 0.05 vs VIT − E HD; data are M ± SD; **, *P* < 0.05 vs VIT − E 60 − min venous HD blood line from samples taken during the same HD session

Figure 1 shows comparison among percent variations of NO formation in all hemodialysis treatments and controls.

## Discussion

In the present study on chronic hemodialysis patients, we have observed elevated IDO1 blood activity and the increase of NO serum levels when matched to healthy individuals. These findings are consistent with published data on uremic patients [20]. Previous results reported that cellulose compared with more biocompatible synthetic membranes such as modified cellulose (e.g. Hemophan®) or polysulphone dialyzers, revealed a lower reactive oxygen species production and higher vitamin E in hemodialysis patients serum levels [35].

The limited number of hemodialysis patients included is a clear study limitation. However, we observed that stratifying our results according to the hemodialysis treatment, VIT–E showed reduced IDO1 blood activity and lower NO serum levels when compared to BHD and HDF patients respectively. Our data also show significantly reduced NO serum levels in VIT–E blood, sampled just after vitamin E–coated membrane surface contact, when compared to BHD and HDF blood samples. It is also relevant to observe that hemodialysis performed by VIT−E was performed as standard low−flux hemodialysis treatment, and solute clearance was not subject to norm as it is in HDF procedures [36]. To the best of our knowledge, this is the first description of IDO1 blood activity and NO serum levels, analysed in standard polysulfone low−flux hemodialysis with or without vitamin E–coated membranes, and HDF.

IDO1 is an ever−present mammalian cytosolic enzyme responsible for catalysing the initial step of Trp catabolism. Trp metabolism has also been identified as a metabolic checkpoint of immuno−regulation, modulating T−cell behaviour including antimicrobial and antitumor defence, antioxidant activity and suppression of autoimmunity. IDO1 activity is increased during hemodialysis, and it is believed that IDO1 expression usually triggers macrophage polarization towards the anti−inflammatory M2 anti−inflammatory phenotype [36]. However, solid data also show that IDO1 cell overexpression decreasing Trp levels may also lead to the production of toxic Kyn metabolites, promoting the release of pro−inflammatory cytokines, polarizing instead macrophages to the M1 pro−inflammatory phenotype [37]. Moreover, a significant link has been found between the increase of Kyn/Trp ratio values and the lowering of vtamin E plasma levels [38]. If there is an IDO1 overexpression in hemodialysis inflammaging, this may explain the coexistence of uremic inflammation and increased immune tolerance. We hypothesize that VIT−E, associated with IDO1 down−regulation, reduces the total number of activated M1− and/or M2−type macrophages, partially adjusting hemodialysis innate immunity dysregulation. Our data show that VIT−E reduces IDO1 blood activity, confirming the acknowledged role vitamin E–loaded dialyzers have in lowering hemodialysis inflammation [7]. It also suggests VIT−E a role has in reducing hemodialysis inflammaging.

Macrophage activation has been reported during the course of hemodialysis, and its triggering results as the main source of mammalian NO. High blood levels of NO modulate not only the innate and adaptive immunity, but also T and B cell differentiation and tumor resistance. High plasma NO levels, have been reported in chronic hemodialysis patients. NO is also produced during hemodialysis, and is known to have a short half−life, as well as showing serum level reduction after the passage of blood through the hemodialysis hemofilter. The local availability of abundant vitamin E anchored to dialysis membrane may have been particularly effective in contrasting dialysis reactive oxygen species generation, and it is important to hypothesize that vitamin E could interfere on hemodialysis macrophage differentiation [38] in VIT−E patients. Our data show that hemodialysis NO plasma formation was significantly reduced after VIT−E treatment when compared to BHD and HDF patients.

Inflammaging is a recently identified immune disorder recognized as a new medical field of human aging, with a growing incidence in metabolic diseases such as type II diabetes and obesity, also related to the growing elderly population worldwide [39]. Aging of the immune system is associated with vaccination resistance, sepsis, and is common in hemodialysis and chronic kidney disease. Immune accelerate aging has instead been observed in chronic inflammation disorders such as HIV, limitating both innate and adaptive immunity [40]. We believe that the accumulation of self−produced toxins in hemodialysis patients, associated with long−term exposure to hemofilter membranes as polysulfone, are highly susceptible to chronic sterile low−grade inflammation, accelerated immunosenescence as well as inflammaging.

IDO1 expression is known to induce the suppression of effector T cells, promoting T_reg_ activation and tolerance. Recently, IDO1 activity has been identified as a potential clinical marker of bacterial infection in hemodialysis patients [41], linking dysregulation of the immune system to hemodialysis chronic microinflammation and risk of infection. Experimental data support the hypothesis that IDO1 suppresses innate and adaptive immune responses, confirming its relevance to promote chronic inflammatory syndromes [42]. Elevated NO levels have also been found in patients with chronic kidney disease [22]. NO is a recognized effector of innate and adaptive immune response, modulating myeloid cell activities, and further promoting chronic microinflammatory disorders [43].

## Conclusions

We hypothesize that hemodialysis microinflammation inducing accelerated immunosenescence and inflammaging could be associated with susceptibility to infections, malignancy and resistance to vaccination. Our data on hemodialysis patients treated with VIT−E, suggest that lowering IDO1 activity and NO formation could partially preserve immune system dysregulation during hemodialysis inflammaging.

## Data Availability

The datasets used and/or analyzed during the current study are available from the corresponding author on reasonable request.

## Abbreviations

BHD: Low−flux bicarbonate hemodialysis
CRP: C−reactive Protein
DAD: Diode array detector
Hb: Hemoglobin
HBV: Hepatitiv B virus
HCV: Hepatitis C virus
HDF: Hemodialfitration
HIV: Human immunodeficiency virus
HPLC: High Performance Liquid Chromatography
IDO1: Indoleamine 2,3-dioxygenase-1
IFN−γ: Interferon−gamma
Kt/V: Dialysis urea clearance normalized for body size
Kyn: Kynurenine
L−Trp: L−tryptophan
NO: Nitric oxide
Trp: Tryptophan
VIT−E: BHD with vitamin E−loaded hemofilters
